# Association Between Hyponatremia and Maintenance Intravenous Solutions in Critically Ill Children: A Retrospective Observational Study

**DOI:** 10.3389/fped.2021.691721

**Published:** 2021-07-06

**Authors:** Jaime Fernández-Sarmiento, Andrea Pérez, Maria Alejandra Echeverri, Paola Jimenez, Maria Alejandra Joachim, Andrés-Jagua

**Affiliations:** ^1^Departament of Pediatrics and Intensive Care, Fundación Cardioinfantil-Instituto de Cardiología, Universidad de La Sabana, Bogotá, Colombia; ^2^Departament of Pediatrics, Fundación Cardioinfantil-Instituto de Cardiología, Universidad El Rosario, Bogotá, Colombia; ^3^Department of Pediatrics and Intensive Care, Universidad de La Sabana, Bogotá, Colombia; ^4^Departament of Research, Universidad Nacional de Colombia, Bogotá, Colombia

**Keywords:** fluids, unbalanced solutions, balanced solutions, sodium, antidiuretic

## Abstract

**Objetive:** We sought to determine the association between maintenance intravenous solutions and the presence of hyponatremia in children in pediatric intensive care (PICU).

**Materials and Methods:** An analytical observational study in children hospitalized in the PICU between January 2015 and December 2018. Patients who received maintenance fluids within the first 48 h after admission and who had at least two serum sodium levels drawn during this time were included.

**Measurements and Main Results:** A total of 1,668 patients were admitted to the PICU during the study period, 503 of whom met the inclusion criteria. The median age was 24 months (IQR 8–96) and 50.9% were female. Altogether, 24.1% of the children developed hyponatremia; it was more frequent in those who received hypotonic solutions (63 vs. 37%; OR 1.41 95% CI 0.92, 2.15 *p* = 0.106), who also had a longer hospital stay (20 vs. 14 days, difference in means 8 days, 95% CI 2.67, 13.3, *p* = 0.001). Children who received loop diuretics and those who were post-operative had a greater risk of developing hyponatremia if they received hypotonic solutions (aOR 2.1 95% CI 1.41, 3.0, *p* = 0.000). Those with balanced isotonic solutions had a lower risk of developing hyponatremia (aOR 0.59 95% CI 0.35, 0.99, *p* = 0.004) and hyperchloremia (aOR 0.51 95% CI 0.34, 0.77, *p* = 0.000), adjusted for disease severity. A greater risk of death was found in the group with severe hyponatremia <130 mEq/L (aOR 9.75 95% CI 1.64–58.15; *p* = 0.01).

**Conclusions:** Hyponatremia associated with the use of hypotonic maintenance solutions occurs in one out of four children in intensive care. The use of these solutions is associated with a longer hospital stay, and the main risk groups are post-operative patients and those who receive loop diuretics. Clinical studies are needed to determine which maintenance solutions have the greatest efficacy and safety in critically ill children.

## Introduction

Maintenance fluids are often used to sustain fluid, metabolic and electrolytic balance in patients who for some reason cannot receive enteral feedings or who only receive partial feedings. The traditional use of intravenous solutions is based on the method proposed by Malcolm Holliday and William Segar in 1957, who, in an effort to approximate the need for solutes and the components of breast milk, proposed hypotonic solutions as maintenance fluids (1–3). The recent American Academy of Pediatrics (AAP) guidelines do not recommend the use of these solutions because they are related to more serious complications which can lengthen hospital stay and cause serious encephalopathy (4).

Hyponatremia has been defined as a serum sodium level of <135 mEq/L. It is the most frequently described fluid and electrolyte complication in hospitalized children (5–8). Various studies have reported a frequency ranging from 19–50% (9). Many of these cases have been associated with the use of excessively hypotonic maintenance fluids, with non-osmotic release of antidiuretic hormone (ADH), especially in risk groups such as patients with respiratory or neurological diseases and those who are post-operative (2, 10, 11). This ADH release may be related to stimuli such as pain, anxiety, fever and vomiting, reducing the capacity to excrete free water, and increasing the risk of hyponatremia in these patients (5, 9, 12).

When the concentration of sodium drops acutely to <125 mEq/L, ~50% of children develop hyponatremic encephalopathy, with possible adverse neurological outcomes (5, 13). Moritz and Ayus described 50 cases of hyponatremia, 26 of whom died of complications related to rapid-onset severe hyponatremia (5).

Over the last few years, several clinical studies and systematic reviews have been published in which the researchers have attempted to clarify which solution is best to use as maintenance fluid. In a recent meta-analysis including more than 2,000 patients, Mercier et al. found that the risk of developing severe hyponatremia (<130 mmol/L) was significantly lower with the use of isotonic solutions compared to hypotonic solutions (RR 0.31; 95% CI 0.19–0.50) (11). Despite this, it has been reported that, in 78% of simulated situations, physicians, including pediatric residents, would prescribe hypotonic maintenance solutions in children (14).

Patients in pediatric critical care have a higher risk of hyponatremia associated with their critical clinical condition and associated comorbidities. An up to four times greater risk of hyponatremia has been reported in the PICU, but it is unclear if this risk changes with the use of the various isotonic solutions available today (balanced or unbalanced) vs. hypotonic solutions (15, 16). In this study, we sought to describe the frequency of hyponatremia in children in intensive care in a reference hospital, and its association with the use of maintenance fluids, as well as the risk factors most often related to this complication.

## Materials and Methods

This was a retrospective cohort observational analytic study of patients from 1 month to 18 years of age who were admitted to the pediatric intensive care unit at the Fundación Cardioinfantil-IC in Bogotá, Colombia (a reference University hospital). Patients hospitalized in the PICU between January 2015 and December 2018 were included. The included children also had to have received maintenance fluids beginning within the first hour after admission to the PICU, have sodium levels within normal limits on admission, and have had a serum sodium level drawn within the first 6 h after admission, with at least two follow up exams within the following 48 h. Only patients receiving a single type of solution as maintenance fluids were included. The same institutional protocol for maintenance fluid solutions was maintained throughout the study period. Patients who received two or more types of intravenous fluids were not included. This study was approved by the institutional ethics committee, with approval number SE-1176-2018. It conformed to the principles of the Declaration of Helsinki, and all patients had a consent form for signed by their parents or legal guardians on admission to the PICU.

Patients with documented hyponatremia on blood gases or serum sodium drawn in the emergency room or hospital wards and processed in the central lab prior to PICU admission were excluded. Those with a diagnosis of adrenal insufficiency, hypothyroidism, syndrome of inappropriate antidiuretic hormone secretion, and/or osmoregulatory readjustment prior to admission were also excluded. The PIM-2 scale was used to evaluate disease severity on admission to the ICU, which was measured in all cases within the first 6 h after admission. Likewise, those who received 3% hypertonic saline due to hyponatremia from other causes were excluded. The patients' admitting diagnosis was considered, and if they required any surgical intervention at any point, they were included as surgical patients for the analysis.

All the data for this study were taken from the electronic medical chart and supported by the clinical laboratory records. All the information was recorded in an exclusive file for this study which had a password known only to the principal investigators. The information obtained from the clinical charts was used exclusively for this study.

Normal saline (NS) or dextrose in normal saline (DNS) were classified as isotonic unbalanced solutions, and Ringer's lactate or Plasmalyte® were classified as balanced isotonic solutions. Two types of hypotonic solutions were used: 80 mEq/L of sodium in 5% dextrose in water (D5W) with 20 mEq of potassium, and 60 mEq/L in D5W with 20 mEq/L of potassium. These solutions are routinely used for maintenance solutions in most middle and low-income countries. Some clinicians especially prefer these hypotonic solutions for the smallest children, to limit the amount of chloride supplied, compared with NS. The volume of fluids administered was calculated according to the institutional protocol using the Holliday-Segar formula, both for medical as well as surgical patients, to cover 100% of their requirements. No fluid restriction was instated for those on mechanical ventilation, or for those recovering from a transplant or any other condition. The only patients who received 70% of the requirement were those who, according to the clinical judgement of the attending physician, were in heart failure and had signs of fluid overload. By institutional protocol, maintenance fluids are reduced to 50% when more than 60% of the caloric intake goals are met through enteral feeding.

Hyponatremia was defined as a serum level <135 mEq/L and hypernatremia as a serum level of more than 145 mEq/L. We defined severe hyponatremia to be serum sodium below 130 mEq/L, after having been admitted with a previously normal level. Potassium and chloride values were also collected, with hypokalemia being defined as potassium <3.5 mEq/L and hyperkalemia as potassium > 5 mEq/L at any point in the measurements during the first 48 h. Likewise, hypochloremia was defined as levels below 97 mEq/L and hyperchloremia as levels above 110 mEq/L in all age groups included in the study.

Serum sodium was measured by the central laboratory, using standard technique, and processing the sample within 1 h of its being drawn, according to the institutional protocol. Samples were sent via pneumatic tube from the PICU, ensuring rapid and effective processing in <60 min. The blood sample was drawn from an arterial line (radial or femoral) or from venous blood drawn from a central venous catheter in the subclavian, jugular or femoral vein. Potentiometry was employed to process the plasma electrolytes, using an Abbott® Architect i8000 system.

The primary outcome was the frequency of hyponatremia associated with the use of intravenous maintenance solutions. Secondary outcomes were length of hospital stay, factors associated with the presence of hyponatremia and mortality.

A univariate analysis was performed describing quantitative variables with central tendency measures, using means and reporting standard deviations assuming normality (according to the Kolmogorov-Smirnov test). For variables with non-normal distribution, medians and interquartile ranges were used. Likewise, qualitative variables were reported using percentages, and the bivariate analysis considered their relationship to the primary outcome. Multivariate analysis was performed to attempt to control for confounding factors that could explain hyponatremia due to another cause. The model included disease severity measured with the PIM-2 scale, age, and a > 10% fluid balance. In addition, variables which met the Hosmer-Lemeshow criteria on the bivariate analysis were considered. The model was constructed using the forward method and was adjusted with the Omnibus test. A *p* < 0.05 was considered to be significant, and STATA 14.0 software was used for the analyses.

## Results

**Figure 1:**
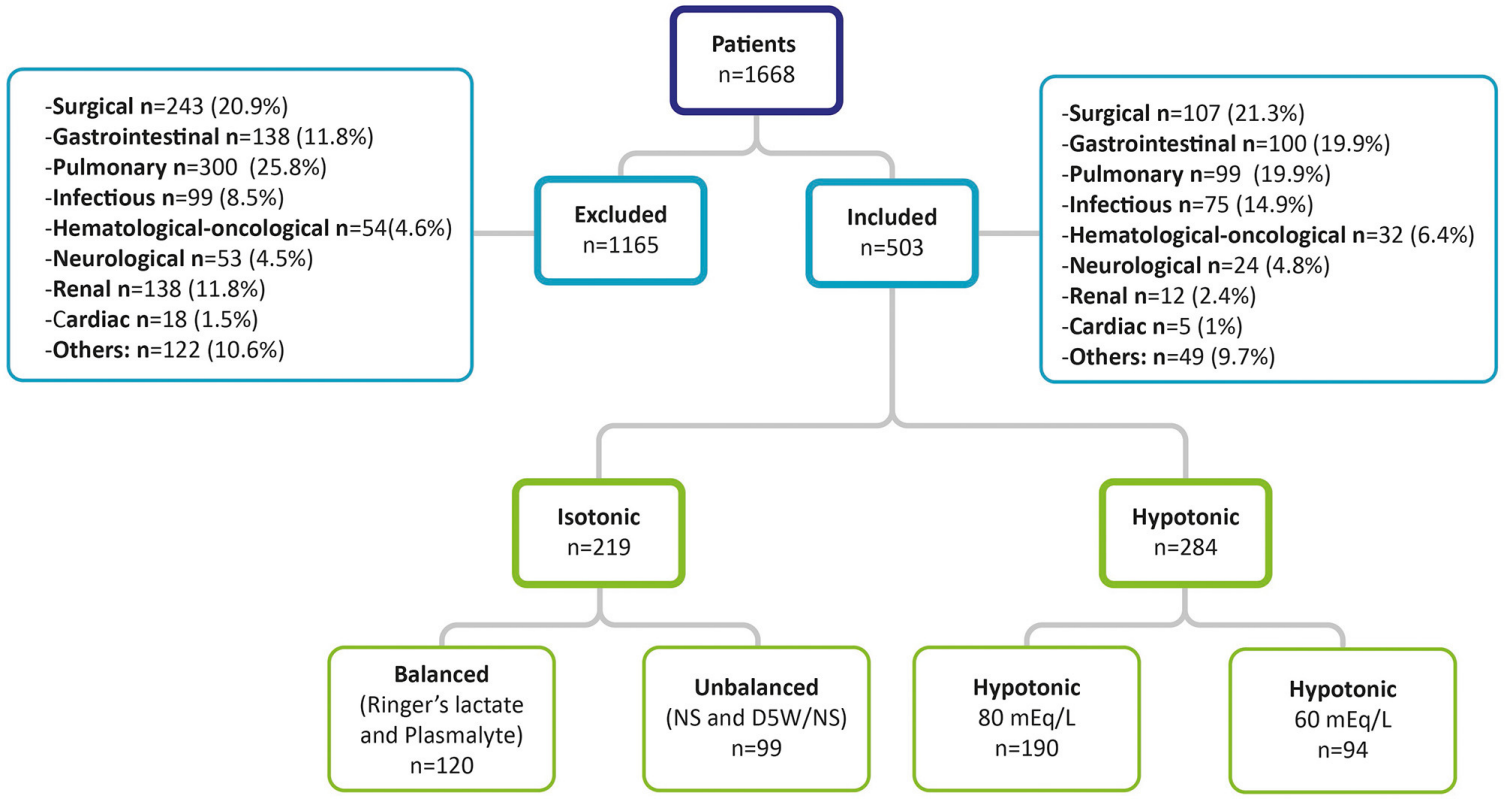
Study population.

During the study period, 1,668 patients were admitted to intensive care. These included 503 children who met the inclusion criteria, 284 (56.5%) of whom received hypotonic solutions and 219 isotonic solutions. Of these, 120 (23.9%) received balanced isotonic solutions (Ringer's lactate or Plasmalyte) and 99 (19.7%) received unbalanced isotonic solutions (NS or D5W/NS). The median age was 24 months for all groups (IQR 8–96 months) and 51% of included patients were female (Figure 1). In addition, hyponatremia was seen in 70 cases (25.2%) of children under the age of two, with no differences found in the frequency of hyponatremia compared with other age groups (*p* = 0.57). Of these patients, 57.3% received a solution with 80 mEq/L of sodium and 23.4% received 60 mEq/L of sodium, with hyponatremia found more often in those under the age of two using these two types of hypotonic solutions (*p* = 0.003) compared with children under the age of two who received an isotonic solution.

Fifty-nine percent of cases were admitted to the PICU for non-surgical conditions (Table 1). There were no significant differences in the distribution of surgical or non-surgical cases by sex or age (*p* = 0.387), nor in disease severity evaluated using the PIM-2 scale, between the groups (*p* = 0.14). The most common causes of hospitalization were the need for pediatric surgery (11.9%), liver transplant (11.7%), and sepsis (11.1%) (Supplementary Table 1). Altogether, 47.3% of the children received mechanical ventilation support for more than 24 h after admission, and 26.8% received vasoactive support for more than 24 h.

**Table 1:** Characteristics of the study population.

		**Isotonic**		**Hypotonic**		
	**Total patients**	**Balanced isotonic solution**	**Unbalanced isotonic solution**	**Hypotonic solution: 80 mEq/L**	**Hypotonic solution: 60 mEq/L**	*P-*value^*^
	**(*n =* 503)**	**(*n =* 120)**	**(*n =* 99)**	**(*n =* 190)**	**(*n =* 94)**	
Female sex, n (%)^**^	256 (50.9)	61 (50.8)	51 (51.5)	90 (47.3)	54 (57.4)	0.954
Age in months, median (IQR)	24 (8–96)	132 (78–168)	72 (12–132)	9 (5–12)^*^	12 (9–36)^*^	0.001
Median length of hospital stay (IQR)	17 (9–34)	13 (8–25)	14 (8–31)	19 (11–36)	22 (10.47)	0.000
Surgical, n (%)	206 (41)	44 (36.7)	38 (38.4)	89 (46.8)	35 (37.2)	0.160
**Reason for hospitalization, n(%)**						
Pulmonary	99 (19.7)	14 (11.7)	14 (14.1)	45 (23.7)	26 (27.7)^*^	0.001
Renal	12 (2.4)	5 (4.2)	3 (3)	1 (0.5)	3 (3.2)	0.156
Cardiac	5 (1)	2 (1.7)	0	1 (0.5)	2 (2.1)	0.347
Infectious	75 (14.9)	13 (10.8)	18 (18.2)	30 (15.8)	14 (14.9)	0.456
Neurological	24 (4.8)	9 (7.5)	4 (4)	34 (17.9)	7 (7.4)	0.756
Hematological-oncological	32 (6.4)	18 (15)^*^	10 (10.1)^*^	2 (1.1)	2 (2.1)	0.001
Gastrointestinal	100 (19.9)	10 (8.3)	11 (11.1)	65 (34.2)	14 (14.9)	0.000
Other	49 (9.7)	20 (16.7)	13 (13.1)	8 (4.2)	8 (8.5)	0.000
**Other diagnoses, n(%)**						
Sepsis	56 (11.1)	8 (6.7)	11 (11.1)	26 (13.7)	11 (11.7)	0.124
Liver transplant	59 (11.7)	5 (4.2)	5 (5.1)	41 (21.6)^*^	8 (8.5)	0.001
**Mechanical ventilation n (%)**	238 (47.3)	47 (39.1)	42 (42.4)	98 (51.6)^*^	51 (54.3)^*^	0.030
**Vasopressors n (%)**	135 (26.8)	36 (30)	22 (22.2)	56 (29.5)	21 (22.3)	0.879
**PIM-2 median, (IQR)**	4.05 (1.3–4.1)	2.57 (1.29–8.60)	4.37 (1.41–8.86)	5.45 (1.19–13.30)	3.42 (1.62–8.35)	0.145
**Use of sedatives n (%)**	248 (49.3)	52 (43.3)	46 (35)	100 (52.6)	50 (53.2)	0.562

* *Chi2-Test Fisher*.

** *The percentage expressed corresponds to the proportion of females in each group*.

Patients hospitalized for pulmonary or gastrointestinal conditions were significantly younger than those hospitalized for other causes (*p* = 0.045). The age group in which hypotonic solutions were used was younger than the age group for whom isotonic solutions were ordered (*p* = 0.001).

Hyponatremia occurred in 121 (24.1%) of the study patients. Thirty-seven percent (45 patients) of the cases occurred with isotonic solutions and 63% (76 patients) with hypotonic solutions. A greater risk of hyponatremia was found with the use of hypotonic solutions compared to isotonic solutions (OR 1.41 95% CI 0.92, 2.15; *p* = 0.106), although the difference was not statistically significant.

When hyponatremia was analyzed by type of solution, balanced isotonic solutions were found to have a lower risk of hyponatremia than the other solutions (aOR 0.59 95% CI 0.35, 0.99, *p* = 0.04), controlling for confounding factors (Table 2). However, there were no differences in the occurrence of hyponatremia with the use of other maintenance solutions: unbalanced isotonic solutions (aOR 1.51 95% CI 0.79, 2.86, *p* = 0.21), hypotonic solution with 80 mEq/L of sodium (aOR 0.73 95% CI 0.40, 1.33, *p* = 0.31), or hypotonic solution with 60 mEq/L of sodium (aOR 1.38 IC95% 0.75, 2.53, *p* = 0.29) Table 2.

**Table 2:** Multivariate model of factors associated with presence of hyponatremia and the type of solution employed^*^.

**Type of solution**	**Risk of hyponatremia** **aOR (95%CI)**	***P*****-value**	**Hospital stay** **aOR (95%CI)**	***P*****-value**	**Mortality** **aOR (95%CI)**	***P*****-value**	**Use of loop diuretics** **aOR (95%CI)**	***P*****-value**	**Postoperative** **aOR (95%CI)**	***P*****-value**
Balanced	0.59 (0.35–0.99)	0.04	0.41 (0.19–0.82)	0.01	0.26 (0.07–1.07)	0.06	0.51 (0.24–1.07)	0.07	0.52 (0.26–1.07)	0.07
Unbalanced	1.51 (0.793–2.86)	0.21	1.11 (0.61–2.01)	0.72	1.13 (0.35–3.68)	0.84	0.89 (0.48–1.65)	0.70	0.99 (0.54–1.79)	0.96
80 mEq/L hypotonic solution	0.73 (0.40–1.33)	0.30	1.44 (0.85–2.43)	0.17	2.72 (0.77–9.62)	0.12	1.52 (1.02–2.41)	0.05	1.30 (1.06–3.06)	0.03
60 mEq/L hypotonic solution	1.38 (0.75–2.53)	0.29	1.08 (0.62–1.90)	0.78	1.05 (0.27–4.04)	0.93	1.82 (1.23–2.53)	0.05	1.5 (1.11–1.82)	0.05

* *Binary logistic regression. aOR (OR adjusted for PIM-2 disease severity, age, fluid balance, need for mechanical ventilation, vasoactives)*.

Similarly, regardless of the type of solution used, post-operative patients were found to have a greater risk of hyponatremia than non-surgical patients (27.4% in the post-operative group vs. 17.1% in the non-surgical group; OR 1.93, 95% CI 1.23, 3.1, *p* = 0.001). The frequency of hyponatremia was even greater if these patients received loop diuretics during the first 48 h after admission (28.3 vs. 18%; *p* = 0.007). The risk of hyponatremia in these patients with the use of diuretics and in the post-operative group increased if they were also receiving hypotonic solutions as maintenance fluids (aOR 2.1 95% CI 1.41, 3.0 *p* = 0.000). This risk increased as the concentration of sodium in the maintenance fluid solution decreased (Table 1). We found no difference in the frequency of hyponatremia if patients received vasoactive support (*p* = 0.87), sedation (*p* = 0.56), mechanical ventilation (*p* = 0.07), or high-flow nasal cannula support (*p* = 0.6).

The frequency of hyponatremia in patients with and without metabolic acidosis was similar (10.1 vs. 20%, OR 2.27 95% CI 0.87–5.65, *p* = 0.085), as well as in children with hyperlactatemia (OR 1.32 95% CI 0.53–3.28, *p* = 0.536) and acute kidney injury (OR 0.51 95% CI 0.15–1.76, *p* = 0.441), regardless of the type of solution used.

Likewise, when the subgroup with severe hyponatremia (<130 mEq/L) was analyzed, we found that there was a greater risk of developing it when solutions with a very low sodium content were used. Specifically, the risk of severe hyponatremia was much greater with the 60 mEq/L solution (aOR 3.87 95% CI 1.32–18.73; *p* = 0.04), and there was a greater risk of death (aOR 9.75 95% CI 1.64–58.15; *p* = 0.01) after adjusting for disease severity.

Hypernatremia was found in 29 patients (5.8%) and no association was found between the frequency of elevated sodium and the type of solution used (*p* = 0.894) or age (*p* = 0.705), nor was it associated with the reason for hospitalization (*p* = 0.621), or the group of post-operative patients (*p* = 0.962). However, if the patient had hypernatremia, there was a greater risk of unsatisfactory clinical progression and greater mortality (25 vs. 4.8%, OR 6.6 95% CI 2.39, 18.22; *p* = 0.001).

The use of balanced isotonic solutions was associated with a lower risk of hyperchloremia (OR 0.51 95% CI 0.34, 0.77, *p* = 0.000). No association was found between chloride disorders and the use of unbalanced and hypotonic solutions (OR 0.74 95% CI 0.47, 1.16, *p* = 0.234) (Supplementary Table 2). No differences were found between the cumulative 48-h balance and the presence of hyponatremia, according to the type of solution employed (*p* = 0.58), but if the cumulative balance was > 10% 48 h after admission, there was a greater risk of developing hyponatremia (OR 2.54 95% CI 1.54–6.03; *p* = 0.013).

**Figure 2:**
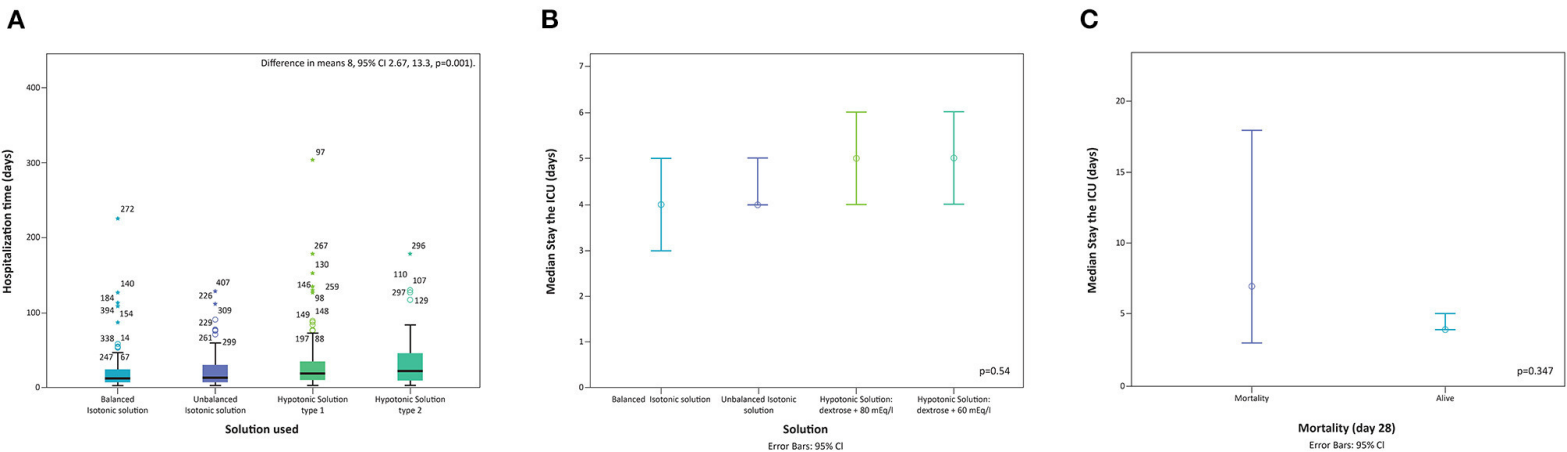
Length of stay in hospital and type of solution used. textbf(A) Distribution of length of hospital stay by solution used. **(B)** Distribution of length of stay in the ICU by solution used. **(C)** Distribution of length of stay in the ICU in mortality cases. Navy blue: Balanced isotonic solution, Purpure: Unbalanced isotonic solution, Dark green: Hypotonic solution type 1, and Light green: Hypotonic solution type 2.

The median length of hospital stay of all the included patients was 17 days (IQR 9–34). The group receiving hypotonic solutions had a longer stay (20 days IQR 11–38) than the group receiving isotonic solutions (14 days IQR 8–30) (*p* = 0.000). The use of hypotonic solutions was associated with 8 more days of total hospital stay (difference in means 8, 95% CI 2.67, 13.3, *p* = 0.001) (Figure 2A). Likewise, after controlling for confounding factors, a shorter hospital stay was found with the use of balanced solutions (aOR 0.41 95% CI 0.19–0.82; *p* = 0.013).

The median ICU stay was 4 days (IQR 3–8) for all groups. There were no differences in mortality between patients receiving hypotonic or isotonic solutions (4.9 vs. 4.6%; *p* = 0.347). There were no differences in length of ICU stay according to the type of solution employed (*p* = 0.543) (Figure 2B).

Hyponatremic encephalopathy was found in four patients (0.8%), with no subsequent neurological damage documented, and recovery with sodium correction.

Death occurred in 24 patients (4.8%) (Figure 2C). Mortality was related to other variables such as sepsis (OR 3.61 95% CI 1.42, 9.42 *p* = 0.011), hypernatremia and lactate > 2 (OR 14.61 95% CI 1.87, 114.21, *p* = 0.001).

## Discussion

The use of intravenous fluids as maintenance solutions is one of the most common practices in hospitalized patients. There is a general consensus regarding the volume administered, based on the formula proposed by Holliday-Segar; however, there is controversy regarding the ideal tonicity, due to reported complications like acute hyponatremia with the use of excessively hypotonic solutions, and the presence of adverse outcomes (13).

This study evaluated 503 patients hospitalized in intensive care who received isotonic or hypotonic solutions as maintenance fluids, finding that 24.1% presented acute hyponatremia within the first 48 h after admission. Of these patients, 37% of cases occurred with the use of isotonic solutions and 63% with hypotonic solutions. This group had a longer hospital stay and hyponatremia was more commonly found in patients on loop diuretics and in post-operative recovery.

The frequency of hyponatremia associated with the use of hypotonic solutions varies according to the study. Carandang et al. (9) evaluated 1,048 patients, among whom, 38.6% of those who received hypotonic solutions developed hyponatremia while 27.8% of those who received isotonic solutions did so (OR 1.63; 95% CI 1.24–2.15, *p* < 0.001). In a group of post-operative children or children with gastroenteritis, Saba et al. (17) found a 20% frequency of hyponatremia associated with the use of hypotonic solutions. However, they found no differences in the frequency of hyponatremia between isotonic or hypotonic solutions. Other researchers have had similar findings (8, 18, 19). The most recent meta-analysis, which included more than 2,000 children, found an overall greater relative risk of developing severe hyponatremia with the use of hypotonic solutions (RR 0.31; 95% CI 0.19–0.50) (11).

Traditionally, the non-osmotic release of ADH has been related to the presence of hyponatremia associated with these solutions (19, 20, 22). Stimuli such as pain, stress and nausea, among others, may trigger this release and favor the presence of hyponatremia when excessively hypotonic solutions are used. Although the superiority of isotonic over hypotonic fluids as maintenance solutions (in terms of hyponatremia prevention) has been described for more than 10 years, unfortunately, ordering excessively hypotonic fluids for hospitalized children, even in the PICU, is still a common practice. The AAP (4) and NICE (20) guidelines recommend always using isotonic solutions as maintenance fluids. However, they do not specify if they should be balanced or unbalanced solutions.

In this regard, our study found that patients who received balanced isotonic solutions had a lower risk of hyponatremia (OR 0.59 95% CI 0.35, 0.99, *p* = 0.007) than those who received unbalanced isotonic solutions as maintenance fluids. Of these, NS with or without 5DW was the solution used in our study. We are unaware of any studies describing this association. It has recently been reported that when NS is used in adults or children as boluses for fluid resuscitation, there is a greater risk of acute kidney injury, hyperchloremia and metabolic acidosis (8, 18, 19). However, in our study we did not find a greater incidence of this type of complications, although it should be clarified that NS was not used in boluses but rather as a maintenance solution. Balanced dextrose-containing solutions are not available in all countries for exclusive maintenance fluid use, especially in small children. Prospective studies are needed to assess the safety and efficacy of the use of balanced solutions vs. unbalanced solutions as maintenance fluids.

Furthermore, most of the studies describe the immediate post-operative period as a risk factor for hyponatremia associated with the use of hypotonic solutions. Carandang et al. (9) found a higher risk of hyponatremia in the subgroup of post-operative children (RR 2.24, 95% CI 1.52–3.31). Choong et al. (21) had similar findings in this group of patients (40.8 vs. 22.7%; RR: 1.82) (95% CI: 1.21–2.74; *p* = 0.004). In our cohort, a greater risk of hyponatremia was also found in the group of post-operative patients (OR 1.93 95% CI 1.23, 3.1, *p* = 0.001). The causes could be related to the administration of anesthetic agents or opioids and the non-osmotic secretion of ADH (22–24). In patients recovering from abdominal surgery, excessive interstitial fluid immediately after the procedure as well as the presence of colostomies (with possible sodium loss) could also explain this association (16).

In addition, the use of balanced isotonic solutions was associated with a lower risk of hyperchloremia (OR 0.51 95% CI 0.34, 0.77 *p* = 0.000), which could be related to the lower supply of chloride in most balanced solutions compared with unbalanced solutions. Hyperchloremia in critically ill children has been associated with the use of chloride-rich solutions mainly administered as volume expanders and maintenance fluids (OR 1.13; 95% CI 1.04–1.23) (25). These may cause complications secondary to renal and splanchnic vasoconstriction, as well as related to increased proinflammatory cytokines, increasing the risk of progressing to multiple organ failure (OR 1.9, 95% CI 1.1–3.2, *p* = 0.023) and greater associated mortality (OR 3.7, 95% CI 2.0–6.8, *p* < 0.001) (26).

Furthermore, we found that patients on loop diuretics had a greater risk of hyponatremia (OR 1.74 95% CI 1.11, 2.73, *p* = 0.007), which was even greater if they were also receiving hypotonic solutions as maintenance fluids (aOR 2.1 95% CI 1.41, 3.0 *p* = 0.000). Although some studies report that 73% of cases of diuretic-induced hyponatremia are due to thiazide diuretics, and only 8% are caused by loop diuretics like furosemide, we found a greater risk of hyponatremia in this group. These diuretics can cause volume depletion, or the patient may receive a high supply of fluids which is poorly tolerated by the children because loop diuretics may partially alter the ability to dilute the urine. These effects may be more frequent in very small children, who also have difficulties in maintaining a hypertonic renal medulla (16, 27).

In our cohort, we also found an average of 8 days' longer hospital stay with the use of hypotonic solutions compared with isotonic solutions (difference in means 8, 95% CI 2.67, 13.3, *p* = 0.001). Shein et al. found that, in patients with bronchiolitis, hyponatremia was correlated with increased hospital stay (r = −0.477, *p* < 0.0001), and thus this may be a modifiable risk factor which could be mitigated by avoiding the use of hypotonic solutions (12).

Outcomes such as death and neurological lesions have been reported as a result of hospital-acquired hyponatremia in children receiving hypotonic intravenous maintenance fluids. Moritz and Ayus found that 9.4% of their patients who had iatrogenic hyponatremia during their hospital stay died, and they suggested a direct relationship between the severity of the hyponatremia and these unsatisfactory outcomes (28). Nevertheless, our cohort had a similar mortality between the groups, except in children with sepsis or hypernatremia (OR 6.6 95% CI 2.39, 18.22, *p* = 0.025) and in children with severe hyponatremia (aOR 9.75 95% CI 1.64–58.15; *p* = 0.01). It is important to carry out studies to determine the risk groups for worse outcomes and their association with the type of solution used as maintenance fluids.

We consider that our study has several limitations. Due to its retrospective nature, the study could be susceptible to information bias; however, the established methodology allowed data capture errors to be controlled, and we used secondary sources (such as nursing notes) when complete data were not available. In addition, the study reflects the experience of a single center which cares for complex patients. Thus, hospitals receiving less complex patients might have less hyponatremia. Altogether, 8.9% of the children who received isotonic solutions developed hyponatremia. Isotonic fluids in the context of ADH elevation may also favor the development of hyponatremia due to free water retention. Children hospitalized in critical care tend to be complex, with multiple comorbidities, many of which may be non-osmotic stimuli for ADH release. Due to the design characteristics of this study, no intervention was performed, and it was not designed to investigate the volume of fluids administered. Therefore, future clinical studies may consider that “restricted” maintenance fluids could be necessary for patients with greater ADH release, thus restricting the supply of free water and, hypothetically, decreasing the frequency of iatrogenic hyponatremia. This is very important, given that a significant proportion of fluid overload in critically ill children is often not recognized by the attending physicians (29).

## Conclusions

The use of maintenance fluid solutions in critically ill children is often associated with a significant incidence of acute hyponatremia within the first 48 h after admission to intensive care. We found that one out of four children receiving maintenance fluids develop acute hyponatremia. This hyponatremia is more frequent and marked in patients receiving hypotonic solutions as maintenance fluids than in those receiving isotonic solutions, and this finding is associated with a more prolonged hospital stay. It is more common in post-operative patients, as well as in children on loop diuretics, groups which have traditionally been considered to be at risk for this type of complication. The use of balanced isotonic solutions as maintenance fluids is associated with a lower frequency of hyponatremia and complications. Clinical studies are needed to evaluate the efficacy and safety of balanced isotonic solutions as maintenance fluids in critically ill children.

## Data Availability Statement

The original contributions presented in the study are included in the article/Supplementary Material, further inquiries can be directed to the corresponding author/s.

## Ethics Statement

The studies involving human participants were reviewed and approved by committee reference number SE-1176-2018, and the parents of the children agreed to participate in this study. Written informed consent to participate in this study was provided by the participants' legal guardian/next of kin.

## Author Contributions

JF-S, AP, ME, PJ, MJ, and A-J contributed to designing and performing the study. AP, ME, PJ, and MJ participated in data collection. JF-S supervised study development and data collection. All the authors contributed to drafting the manuscript and reviewing the final article. All authors approved the final manuscript as submitted and agree to be accountable for all aspects of the work.

## Conflict of Interest

The authors declare that the research was conducted in the absence of any commercial or financial relationships that could be construed as a potential conflict of interest.
