# Supplementary material for: Association Between Hyponatremia and Maintenance Intravenous Solutions in Critically Ill Children: A Retrospective Observational Study
Source: Front Pediatr. 2021 Jul 6;9:691721. doi: 10.3389/fped.2021.691721 (PMC8290911; doi:10.3389/fped.2021.691721)
Supplement: Supplementary file 1 [file Table_1.docx]

**Supplement 1. Distribution of reasons for hospitalization, grouped by systems, on admission to the Critical Care Unit.***

|  | **Frequency** | **%** |
| --- | --- | --- |
| **Surgical** | 107 | 21.3 |
| **Gastrointestinal** | 100 | 19.9 |
| **Pulmonary** | 99 | 19.7 |
| **Infectious** | 75 | 14.9 |
| **Other** | 49 | 9.7 |
| **Hematological- oncological** | 32 | 6.4 |
| **Neurological** | 24 | 4.8 |
| **Renal** | 12 | 2.4 |
| **Cardiac** | 5 | 1.0 |
