# Supplementary material for: Association Between Hyponatremia and Maintenance Intravenous Solutions in Critically Ill Children: A Retrospective Observational Study
Source: Front Pediatr. 2021 Jul 6;9:691721. doi: 10.3389/fped.2021.691721 (PMC8290911; doi:10.3389/fped.2021.691721)
Supplement: Supplementary file 2 [file Table_2.DOCX]

**Table 2. Type of solution and association with hyperchloremia**

| **Type of solution** | | **OR** | **95% CI** | **P value** |
| --- | --- | --- | --- | --- |
| **Isotonic solution** | **Balanced** | 0.51 | 0.34-0.77 | 0.000 |
|  | **Unbalanced** | 0.74 | 0.47-1.16 | 0.234 |
| **Hypotonic solution** | **80 mEq/L Na** | 1.79 | 1.23-2.62 | 0.000 |
|  | **60 mEq/L Na** | 1.32 | 0.82-2.11 | 0.242 |
